# Non-end-to-end adaptive graph learning for multi-scale temporal traffic flow prediction

**DOI:** 10.1371/journal.pone.0322145

**Published:** 2025-06-11

**Authors:** Kang Xu, Bin Pan, MingXin Zhang, Xuan Zhang, XiaoYu Hou, JingXian Yu, ZhiZhu Lu, Xiao Zeng, QingQing Jia

**Affiliations:** 1 School of Artificial Intelligence and Software, LiaoNing Petrochemical University, Fushun, China; 2 College of Science, LiaoNing Petrochemical University, Fushun, China; 3 College of Information and Control Engineering, LiaoNing Petrochemical University, Fushun, China; 4 Northeast Forestry University, Heilongjiang, China; University of Essex Faculty of Science and Engineering, UNITED KINGDOM OF GREAT BRITAIN AND NORTHERN IRELAND

## Abstract

Accurate traffic flow prediction is vital for intelligent transportation systems but presents significant challenges. Existing methods, however, have the following limitations: (1) insufficient exploration of interactions across different temporal scales, which restricts effective future flow prediction; (2) reliance on predefined graph structures in graph neural networks, making it challenging to accurately model the spatial relationships in complex road networks; and (3) end-to-end training, which often results in unclear optimization directions for model parameters, thereby limiting improvements in predictive performance. To address these issues, this paper proposes a non-end-to-end adaptive graph learning algorithm capable of effectively capturing complex dependencies. The method incorporates a multi-scale temporal attention module and a multi-scale temporal convolution module to extract multi-scale information. Additionally, a novel graph learning module is designed to adaptively capture potential correlations between nodes during training. The parameters of the prediction and graph learning modules are alternately optimized, ensuring global performance improvement under locally optimal conditions. Furthermore, the graph structure is dynamically updated using a weighted summation approach.Experiments demonstrate that the proposed method significantly improves prediction accuracy on the PeMSD4 and PeMSD8 datasets. Ablation studies further validate the effectiveness of each module, and the rationality of the graph structures generated by the graph learning module is visually confirmed, showcasing excellent predictive performance.

## Introduction

With the rapid development of cities, the growing number of vehicles and the insufficient urban traffic capacity have become increasingly contradictory. Intelligent Transportation Systems (ITS) are one of the key technologies to address this issue [1]. Traffic flow prediction is an indispensable component of intelligent transportation systems, particularly on highways with high traffic volumes and fast-moving vehicles. Due to the relatively closed nature of highways, congestion, once it occurs, can severely impact traffic capacity. If traffic flow can be accurately predicted in advance, traffic management authorities can better guide vehicles and improve the operational efficiency of the highway network.

**Fig 1:**
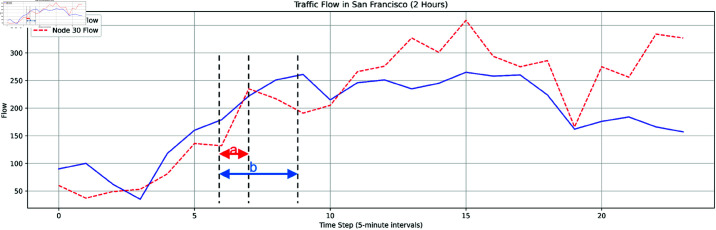
Traffic flow chart of Node 10 and Node 30.

Traffic flow prediction is a spatio-temporal forecasting task. On a macroscopic level, traffic data usually exhibit similar patterns, whether on a short-term or long-term time scale. For example, dynamic congestion occurs during peak hours, and there are stable traffic pattern differences between weekdays and weekends. As illustrated in Fig 1, the congestion period b at node 10 is similar in congestion intensity to the congestion period a at node 30, though their durations vary. Clearly, *a* and *b* are at different time scales, but their traffic patterns are similar. However, many models based on Recurrent Neural Networks (RNNs) or temporal attention mechanisms (e.g., DCRNN [2], GMAN [3]) rely solely on the interaction of spatio-temporal features of the same time length to predict future traffic conditions, making it difficult to capture the correlation between spatio-temporal features of similar traffic patterns at different time scales [4–6].

**Fig 2:**
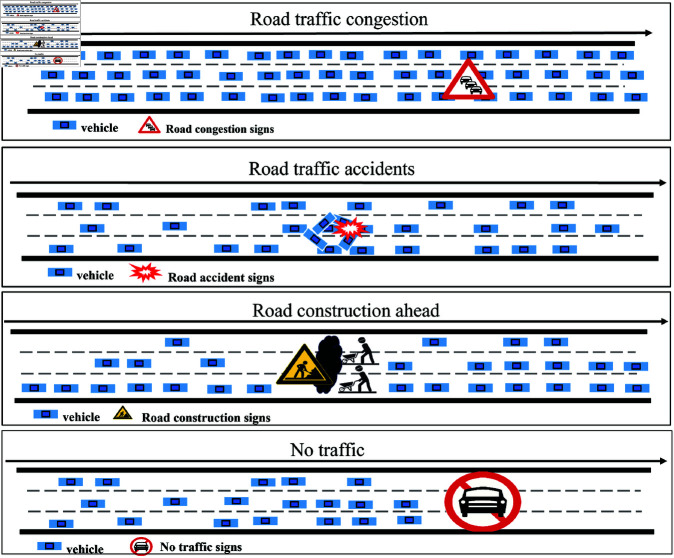
Traffic incidents on highways.

On a microscopic level, as shown in Fig 2, due to the complex interactions between numerous unique and random traffic events (e.g., congestion, accidents, construction, road closures, etc.), traffic data in the same region exhibit dynamic and complex fluctuations [7,8]. Therefore, effectively extracting the spatio-temporal features of traffic flow has become a crucial step in traffic flow prediction.

**Fig 3:**
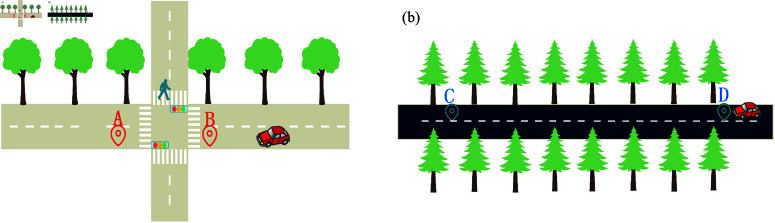
(a) Although A and B are close to each other, they weakly depend on one another due to the presence of a crossroad between them. (b) Although C and D are far apart, they strongly interdepend as they are located on the same highway.

The complexity of road networks and the instability of connections between nodes have led to many networks experiencing bottlenecks in recent years [9,10]. This issue arises because predefined adjacency matrices are mostly created based on expert judgment. For instance, Fig 3 illustrates this: in Fig 3, although nodes A and B are geographically close, their traffic flow relationship is altered by the presence of traffic lights. Conversely, in Fig 3b, nodes C and D, despite being further apart, typically share a similar traffic flow. Consequently, relying on predefined rules to define the relationships between traffic nodes is often inaccurate [11]. This approach impedes the extraction of hidden spatial dependencies in traffic data, as these relationships should be discovered from actual data rather than being assumed as prior knowledge.

Currently, most spatio-temporal graph neural networks use an end-to-end training approach, where the backbone network is usually split into a graph learning module and a prediction module [12]. The effectiveness of the prediction module’s adaptive training heavily relies on the graph learning module’s performance [13]. However, because these two modules often do not stay in an optimal state throughout the training process, it becomes difficult to precisely control the direction of parameter updates, making it challenging to achieve optimal results.

To tackle the aforementioned issues, this paper introduces a non-end-to-end adaptive graph learning algorithm, with the following key contributions:

1. Design of Multi-Scale Temporal Attention and Multi-Scale Temporal Convolution Components: The multi-scale temporal attention module uses a pyramid architecture to capture both coarse-grained and fine-grained temporal information, thereby enhancing the model’s capability to learn features across multiple time scales. Meanwhile, the multi-scale temporal convolution component is designed to capture information from various time scales concurrently, addressing the limitation of traditional temporal convolutions that are restricted to a single time scale.
2. Design of the Adaptive Graph Learning Module: This module constructs a self-learning graph by leveraging actual road data, replacing predefined fixed graph structures. This approach allows the model to more accurately capture the complex relationships between roads.
3. Non-end-to-end training framework: The network framework adopts a non-end-to-end training approach, where the prediction module focuses on extracting temporal features, and the graph learning module focuses on extracting spatial features. This approach allows each module to optimize its own task, reducing mutual interference and improving prediction accuracy.

## Related work

Traffic flow prediction, a central challenge in intelligent transportation systems for smart cities, has attracted significant attention from researchers. In earlier studies, statistical models commonly employed for traffic flow [18,19] prediction included HA [14], ARIMA [15], and VAR [13]. However, these models often struggle with real-world tasks due to the complexity and dynamic nature of traffic flow. While traditional machine learning models like LSTM [16] and SVR [17] can handle more complex data and typically outperform statistical models, they demand meticulous feature engineering, which is both time-consuming and labor-intensive.

In recent years, deep learning methods have been extensively applied to various spatio–temporal prediction tasks, thanks to their powerful learning capabilities. These methods are primarily categorized into two types: grid-based and graph-based approaches. Grid-based methods partition the study area into regular grids, utilizing convolutional neural networks to extract spatial features and recurrent neural networks to manage temporal dependencies in time-series data. However, this approach often neglects the topology of the road network and struggles to accurately represent the spatial relationships between different areas. In contrast, graph-based methods leverage the topology of the road network to construct a graph, effectively capturing the hidden spatial relationships.

Most existing spatio-temporal graph neural networks use predefined graphs, assuming that the potential relationships between nodes are predetermined. However, due to incomplete data connections, this graph structure is unable to accurately reflect the actual dependencies, potentially leading to the loss of real relationships. Therefore, some researchers use graph learning modules to dynamically obtain the graph structure. For example, Graph WaveNet [20] obtains more reliable bidirectional relationships between nodes through an adaptive graph module; DDSTGCN [21] uses a dual hypergraph to capture hidden relationships in the graph. However, this method lacks the guidance of prior knowledge, which makes it susceptible to overfitting or underfitting problems.

## Materials and methods

**Fig 4:**
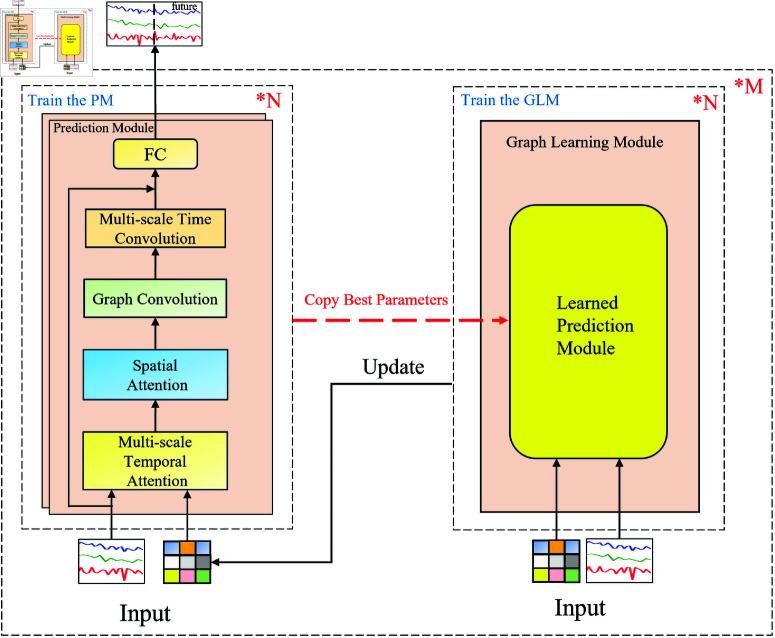
Model architecture overview.

The backbone network proposed in this paper is shown in Fig 4, consisting of a prediction module and a graph learning module. The prediction module is composed of a multi-scale temporal attention component, a spatial attention component, a graph convolution component, and a multi-scale temporal convolution component. The model adopts a non-end-to-end training method: when the prediction module on the left reaches an optimal state within *N* rounds, its optimal parameters are copied to the trained prediction module in the graph learning module on the right, enabling the graph learning module to focus on generating a graph that better fits the real traffic flow data. After each training round, the graph learning module outputs the generated graph to the graph update module and passes it to the trained prediction module on the right to continue training. After the graph learning module completes *N* rounds of training, it outputs a weighted graph, which is then used as the input for the prediction module on the left.

### Multi-scale temporal attention component

First, the original self-attention mechanism is introduced. Let the input sequence be *X*, and the sequence after the attention mechanism output is *Y*. The self-attention mechanism normalizes by column using the softmax function, as shown below:

$$Q=XW_{Q}$$ (1)

$$K=XW_{K}$$ (2)

$$V=XW_{V}$$ (3)

$$Y=V\left(\text{softmax}\left(\frac{K^{T}Q}{\sqrt{D_{K}}}\right)\right)$$ (4)

which *Q*, *K* and *V* represent the query, key, and value sequences, respectively, and $W_{Q},W_{K},W_{V}\in {\mathbb{R}}^{L\times D_{K}}$ are the corresponding linear transformation weight matrices. *L* represents the sequence length, and *D*_*K*_ is the matrix dimension.

**Fig 5:**
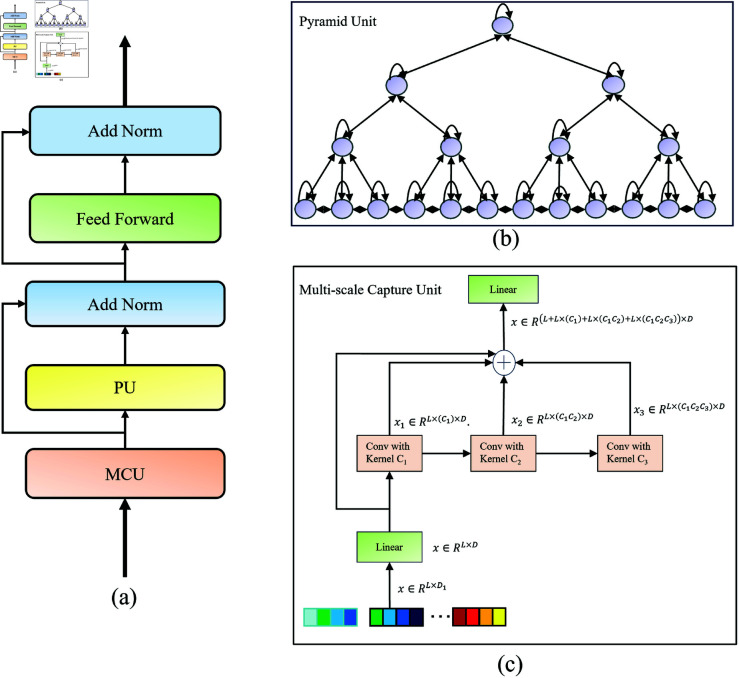
Multi-scale temporal attention component.

Since traffic flow data contains multi-scale information, and the original attention mechanism struggles to capture multi-scale temporal information, this paper adopts a multi-scale temporal attention component to extract multi-scale temporal features. The structure of this component is illustrated in Fig 5a. To enable information transmission in the subsequent pyramid unit (PU) nodes, the required node information for the PU is first generated through the multi-scale extraction unit (MCU). As shown in Fig 5b, first, given the input sequence $𝐗\in {\mathbb{R}}^{L\times D_{1}}$, where L is the length of the sequence, and *D*_1_ and D are the dimensions of the nodes before and after the linear layer, a single linear layer is applied for transformation:

$${𝐗}_{L}\in {\mathbb{R}}^{L\times D}=𝐗\cdot {𝐖}_{1}$$ (5)

Then, the transformed ${𝐗}_{L}\in {\mathbb{R}}^{L\times D}$ is passed through convolutional layer 1 with a kernel size and stride of *c*_1_, convolutional layer 2 with a kernel size and stride of *c*_2_, and convolutional layer 3 with a kernel size and stride of *c*_3_, to extract features at different scales of the time series data, as follows:

$${𝐗}_{1}\in {\mathbb{R}}^{L/(c_{1})\times D}=Conv({𝐗}_{L})$$ (6)

$${𝐗}_{2}\in {\mathbb{R}}^{L/(c_{1}c_{2})\times D}=Conv({𝐗}_{1})$$ (7)

$${𝐗}_{3}\in {\mathbb{R}}^{\frac{L}{c_{1}c_{2}c_{3}}\times D}=Conv({𝐗}_{2})$$ (8)

As shown, at the *S* scale, a sequence of length approximately *L*/*C*^*S*^ is generated, which reduces the data length and thus does not significantly increase the time complexity. Furthermore, by performing convolution on the corresponding sub-nodes, the receptive field of the layer nodes is gradually expanded from bottom to top, enabling the upper-layer nodes to capture a larger range of temporal information. Then, the sequences at the three scales and the input sequence are concatenated along the temporal dimension and further transformed through a linear layer:

$${𝐗}_{P}\in {\mathbb{R}}^{M\times D}=Concat({𝐗}_{L},{𝐗}_{1},{𝐗}_{2},{𝐗}_{3})$$ (9)

$${𝐗}_{O}={𝐗}_{P}\cdot {𝐖}_{2}$$ (10)

The output of the above MCU, ${𝐗}_{O}\in {\mathbb{R}}^{M\times D}$, can be obtained as the input to the PU. Where $M=(L+L/(c_{1})+L/(c_{1}c_{2})+L/(c_{1}c_{2}c_{3}))$.

As shown in Fig 5c, the PU can be structurally decomposed into two parts: inter-scale connections and intra-scale connections.

The inter-scale connections form a tree structure, distributed from top to bottom. Each node from the first to the third layer has $n_{1},n_{2}$ and *n*_3_ child nodes, respectively. An $n_{1}-ary$ tree is formed between the first and second layers, where each parent node has *n*_1_ child nodes, and each parent node is directly connected to *n*_1_ child nodes. The inter-scale structure contains 3 sets of convolution layers, with the kernel sizes and strides from the first to the fourth layers being $c_{1},c_{2}$ and *c*_3_, respectively.

In the intra-scale connections, the window size is set to *S* (in Fig 5, let *S* = 3), meaning that each node is connected not only to itself but also directly to the previous and next nodes, ensuring symmetric connections in both directions. Of course, for the first node (or the last node) at the same scale, it is only directly connected to the second node (or the second-to-last node) in addition to being connected to itself.

Therefore, for node $n_{i}^{\left(s\right)}$, its adjacency set *N*(*i*) is:

$$N(i)=A_{i}^{\left(s\right)}\cup C_{i}^{\left(s\right)}\cup P_{i}^{\left(s\right)}$$ (11)

Here, the adjacent node set $A_{i}^{\left(s\right)}$ refers to the nodes located at the same scale *S* and adjacent in time, with a total of *A* nodes. The child node set $C_{i}^{\left(s\right)}$ represents the *C* child nodes of node $n_{i}^{\left(s\right)}$ in the finer scale *S*–1. The parent node set $P_{i}^{\left(s\right)}$ refers to a parent node of node $n_{i}^{\left(s\right)}$ in the coarser scale S+1.

By calculating the output *Y* from the input *X* of the PU, the sequence *Y* is finally obtained after being processed by the PU.

$$𝐀\in {\mathbb{R}}^{M\times M}=\left(\frac{𝐐{𝐊}^{T}}{\sqrt{d_{k}}}\right)$$ (12)

𝐀=𝐀⊙mask (13)

$$𝐘\in {\mathbb{R}}^{M\times D}=\text{softmax}(𝐀)\cdot 𝐕$$ (14)

The Mask matrix is employed to mask out the node pairs that are not connected. The final output of the multi-scale time attention module is $𝐘\in {\mathbb{R}}^{M\times D}$, where *M* represents the total length of the multi-scale time series, and thus, the first *L* time steps are selected for output.

In conclusion, the multi-scale temporal attention module improves the ability to capture multi-scale information by utilizing local connections and compressed representations of long-range dependencies in the pyramid structure.

### Multi-scale temporal convolution component

**Fig 6:**
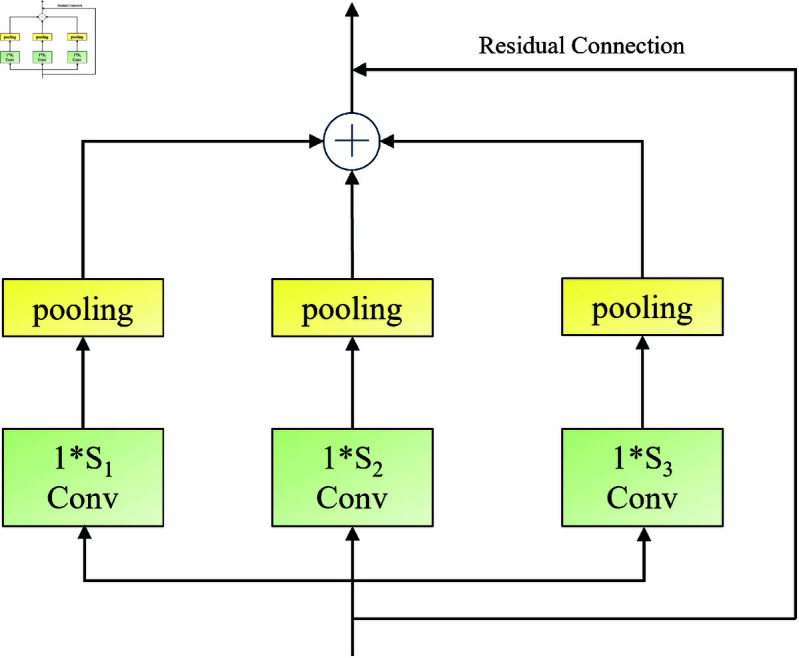
Multi-scale time convolution component.

Traditional temporal convolution components only have a single receptive field, making it difficult to effectively capture multi-scale temporal information in traffic flow data. To better extract multi-scale temporal information, this paper introduces a novel multi-scale temporal convolution component to capture dynamic information over time. As shown in Fig 6, the component mainly consists of three gated units with different receptive fields.

First, introduce the original temporal convolution component:

$${𝒵}^{\prime (l)}=\Gamma *{𝒵}^{\left(l\right)}=\phi (A)⊙\sigma (B)$$ (15)

where ${𝒵}^{\prime (l)}$ is the output of the temporal convolution unit, Γ is the convolution kernel, ${*}_{\tau }$ is the gated convolution operator, ${𝒵}^{\left(l\right)}$ is the input, ϕ(·) is the tanh function, and σ(·) is the sigmoid function. *A* and *B* correspond to the first and second halves of the channel dimension of ${𝒵}^{\prime (l)}$, respectively.

$$Z_{out}^{\left(l\right)}=MTCN({𝒵}^{\prime (l)})=\;ReLU(Concat\left(Pooling\left(\Gamma_{1}{*}_{\tau }{𝒵}^{\prime (l)}\right)\right),\;Pooling(\Gamma_{2}{*}_{\tau }{𝒵}^{\prime (l)}),\;Pooling(\Gamma_{3}{*}_{\tau }{𝒵}^{\prime (l)}))+{𝒵}^{\prime (l)})$$ (16)

where *MTCN*() indicates that the model input is processed through the Multi-scale Time Convolution Component. $\Gamma_{1},\Gamma_{2}$ and $\Gamma_{3}$ are convolution kernels with sizes of $1\;*\;S_{1},1\;*\;S_{2}$ and $1\;*\;S_{3}$, respectively. After applying a pooling operation with a window size *W*, the output dimensions of the three temporal convolution units become


$$\frac{M-(S_{1}-1)}{W},\frac{M-(S_{2}-1)}{W},\frac{M-(S_{3}-1)}{W}.$$

The *Concat*() operation connects the outputs of the three temporal convolution units at different scales, resulting in a feature dimension of $(\begin{array}{c} 3M-(S_{1}+S_{2}+S_{3}-3) \end{array})/W$. In this part, the hyperparameters *S*_1_, *S*_2_, *S*_3_ and *W* can be adjusted to ensure that the output size equals the input size, i.e., $(\begin{array}{c} 3M-(S_{1}+S_{2}+S_{3}-3) \end{array})/W=M$. This allows the use of skip connections, and finally, the output $Z_{out}^{\left(l\right)}\in {\mathbb{R}}^{N\times M\times C^{\left(l\right)}}$ is obtained through the *ReLU* activation function. The multi-scale temporal convolution module effectively reduces gradient dispersion and maintains nonlinearity by utilizing temporal convolution units and residual structures.

### Graph learning module

From a broader perspective, the spatial relationships between nodes in a graph are generally stable, reflecting their inherent associations. However, a predefined adjacency matrix typically captures only basic proximity and fails to effectively extract more complex similarities and relationships between nodes. Consequently, it overlooks valuable information, as predefined rules often miss certain implicit relationships related to traffic conditions [22]. To address this issue, we propose a method to learn these implicit relationships, which are difficult to capture with predefined rules. This approach enhances the representational capacity of the adjacency matrix, thereby more comprehensively capturing the relationships between nodes. The specific process for obtaining this enhanced adjacency matrix is outlined as follows:

First, initializing the current adjacency matrix is critical to the model presented in this paper. The accuracy and reliability of the initial associations between nodes significantly influence the module’s optimization. As a result, random initialization is not suitable; instead, prior information should be employed to construct a set of adjacency matrices that encompass different types of spatial dependencies.

The following formula represents the re-normalization process of the adjacency matrix *A*_*k*_, which ensures the comparability of different types of affinity matrices.

$$A_{k}⇐{\tilde{D}}_{k}^{-\frac{1}{2}}{\tilde{A}}_{k}{\tilde{D}}_{k}^{-\frac{1}{2}}$$ (17)

Equations (18) and (19) describe the renormalization technique, which ensures that different types of adjacency matrices can be compared at the same scale.

$${\tilde{A}}_{k}=A_{k}+I_{N}$$ (18)

$${\tilde{D}}_{k}^{\left(ii\right)}=\sum_{j}{\tilde{A}}_{k}^{\left(ij\right)}$$ (19)

Based on Eq (20), the final composite adjacency matrix *A* can be calculated by combining multiple types of adjacency matrices. It is generated by taking a weighted average of the positions of each adjacency matrix.

$$A^{\left(i,j\right)}=\frac{\sum_{k=1}^{N_{r}}A_{k}^{\left(i,j\right)}}{\sum_{k=1}^{N_{r}}I\left[A_{k}^{\left(i,j\right)}\right]}$$ (20)

$$M(x)=\left\{ \begin{array}{cc} 1 & \;if\;x\neq 0\\ 0 & \;else \end{array}\right.$$ (21)

Next, a coarse adjacency matrix is generated to capture the hidden relationships:

$$A_{1}=ReLU(M_{1}M_{2}^{T}-M_{2}M_{1}^{T})+Diag(\Lambda )$$ (22)

where *M*_1_ , *M*_2_ and Λ are learnable parameters. First, a skew-symmetric matrix is constructed using $\left(M_{1}M_{2}^{T}-M_{2}M_{1}^{T}\right)$. Then, the *ReLU* activation function is applied to set half of the diagonal and other positions to zero. Next, Diag(Λ) is used to generate weights for the diagonal positions. These parameters are summed to obtain $A_{1}$, the parameterized adjacency matrix. Subsequently, the adaptive aggregation module from equations (23) and (24) combines the original adjacency matrix with the new parameterized adjacency matrix to form a new adjacency matrix.

$$S_{attn}=\sigma \left(W\times \left[\begin{array}{c} A_{1}\\ A_{old} \end{array}\right]+b\right)$$ (23)

$$A_{2}=S_{attn}⊙A_{1}+(1-S_{attn})⊙A_{old}$$ (24)

The weight matrix $S_{\text{attn}}$ is used to perform a weighted operation on the original matrix and the coarse affinity matrix to obtain *A*_2_, where ⊙ represents element-wise multiplication.

The proposed graph learning module ensures that the generated adjacency matrix maintains sparsity, enhancing training efficiency and better highlighting significant relationships between nodes while ignoring less important ones. The spatial dependencies in the new adjacency matrix are built upon the previous one, allowing both initial and adaptively learned information to be continuously utilized during iterations, which aids in accelerating convergence.

After *N* round of training in the graph learning module, the resulting adjacency matrix needs to be output as a graph for further training in the prediction module. To capture the hidden relationships in the current road network, the adjacency matrix used in the prediction module is derived by weighting the importance of different subgraphs in the adjacency matrix set *A*. Initially, each subgraph from set *A* is input into the prediction module along with the validation set to compute the corresponding prediction loss, defined as:

$$L_{k}=L_{P}[P(X|A_{k},\theta ),Y]$$ (25)

$$L_{P}(Y,\hat{Y})=∥Y-\hat{Y}{∥}_{1}$$ (26)

where *L*_*P*_ represents the L1 loss, serving as a fundamental metric for assessing the model’s predictive performance. *Y* denotes the true values, and $\hat{Y}$ represents the predicted values. *P*() is the prediction function, and θ are the learnable parameters of the prediction module. The vector $\vec{l}={(}^{\begin{array}{c} L_{1},L_{2},\ldots ,L_{N_{r}} \end{array})T}$ consists of all the prediction losses.$L_{max}=\max_{1\leq i\leq N_{r}}L_{i}$ is the maximum value, and the weight vector $w=\left(w_{1},w_{2},...,w_{N_{r}}\right){\;}^{T}$ can be defined as:

$$w=softmax(L_{max}-\vec{l})$$ (27)

The adjacency matrix used in the prediction module is the weighted sum update of all current subgraphs, i.e.,

$$A=\sum_{i=1}^{N_{r}}w_{i}A_{i}$$ (28)

## Experiments

### Dataset

In this paper, the PeMSD4 and PeMSD8 traffic datasets are used for model validation, both of which were proposed by STSGCN [23]. The time interval for each dataset is 5 minutes, so each hour contains 12 time intervals. Detailed statistics and descriptions of the datasets are shown in Table 1.

**Table 1:** The statistics of various datasets.

Dataset	Sensor	Timesteps	Time Range
PeMSD4	307	16992	01/2018–02/2018
PeMSD8	170	17856	07/2016–08/2016

### Evaluation metrics

This paper uses three effectiveness metrics to assess the accuracy of the model: Root Mean Square Error (RMSE), Mean Absolute Error (MAE), and Mean Absolute Percentage Error (MAPE). The definitions of these metrics are given in (Eqs 29-31).

$$RMSE=\sqrt{\frac{1}{M}\sum_{i=1}^{M}(y_{i}-{\hat{y}}_{i}{)}^{2}}$$ (29)

$$MAE=\frac{1}{M}\sum_{i=1}^{M}|y_{i}-{\hat{y}}_{i}|$$ (30)

$$MAPE=\frac{1}{M}\sum_{i=1}^{M}\left|\frac{{\hat{y}}_{i}-y_{i}}{y_{i}}\right|$$ (31)

where *y*_*i*_ represents the true value, *y*_*i*_ represents the predicted value, and *M* is the number of test samples. RMSE is sensitive to large or small values and can be used to analyze the stability of the predictions; MAE measures the overall error of the predictions and is less affected by outliers; MAPE reflects the degree of bias but is sensitive to the magnitude of the true values.

### Baseline models

The proposed model is compared to 12 advanced benchmark models, as outlined below:

- HA [11]: Traffic conditions are first regarded as time series data with distinct seasonal patterns. By analyzing historical data, average traffic flow or other relevant metrics for different time periods can be calculated. These historical averages are then used to predict future traffic conditions. However, this approach is limited by its oversimplification of traffic complexity, ignoring potential variations and anomalies.
- ARIMA [12]: It effectively captures trends, seasonality, and cyclic changes in time series data for accurate forecasting. Typically, by analyzing the data’s autocorrelation and partial autocorrelation, appropriate ARIMA model orders can be selected to fit the data and make predictions.
- VAR [10]: This is a powerful time series analysis tool, especially suitable for handling complex relationships and correlations among multiple variables. By using the VAR model, the characteristics and dynamic changes of multivariate time series data can be better understood, enhancing the accuracy of predictions and analyses.
- SVR [14]: As the regression form of support vector machines, it possesses strong fitting and generalization capabilities for tasks like traffic forecasting.
- LSTM [13]: As an improved version of RNNs, LSTM excels in long-term time series forecasting. With its gating mechanisms and memory cells, it effectively captures long-term dependencies, making it a powerful tool for complex time series prediction tasks.
- GWN [15]: An effective deep spatiotemporal graph modeling method, GWN leverages adaptive adjacency matrices and node embeddings to learn spatial relationships while using dilated convolutions to capture temporal dependencies. Despite its advantages in handling spatiotemporal graph data, attention must be paid to challenges such as computational complexity and parameter tuning.
- ASTGCN [24]: Employing attention mechanisms, ASTGCN models spatiotemporal dynamics in traffic data through CNNs and GCNs, effectively integrating spatial and temporal information. It is suitable for traffic data forecasting and analysis tasks.
- STSGCN [23]: Utilizing spatiotemporal synchronous techniques to extract local spatiotemporal correlations, STSGCN is well-suited for handling spatiotemporal data. It effectively integrates spatial and temporal information, enhancing performance in spatiotemporal data modeling tasks.
- STFGNN [25]: By designing dynamic time-warped temporal graphs, STFGNN focuses on feature-aware spatial relationships, offering a robust spatiotemporal data modeling approach. It has significant advantages in handling complex spatiotemporal dynamics and functional relationships.
- STGODE [26]: Combining GCN with neural ODE, STGODE provides an effective mechanism to alleviate the over-smoothing problem while capturing complex temporal and spatial dynamics in spatiotemporal data.

### Experimental setup

Experiments were conducted on a machine equipped with an NVIDIA RTX 4090 and 24GB memory, running Ubuntu 20.04. Our model utilized PyTorch 2.0.0 and CUDA 11.8, with the Adam optimizer employed during training. The PeMSD4 and PeMSD8 datasets were split into training, validation, and test sets in a 6:2:2 ratio. To ensure training stability, we applied z-score normalization, as detailed in Eq (32).

$$\hat{x}=\frac{x\;-\;mean(x_{trian})}{std(x_{train})}$$ (32)

where *mean*() and *std*() represent the mean and standard deviation functions, and *x*_*train*_ refers to the training set.

### Performance comparison table

**Fig 7:**
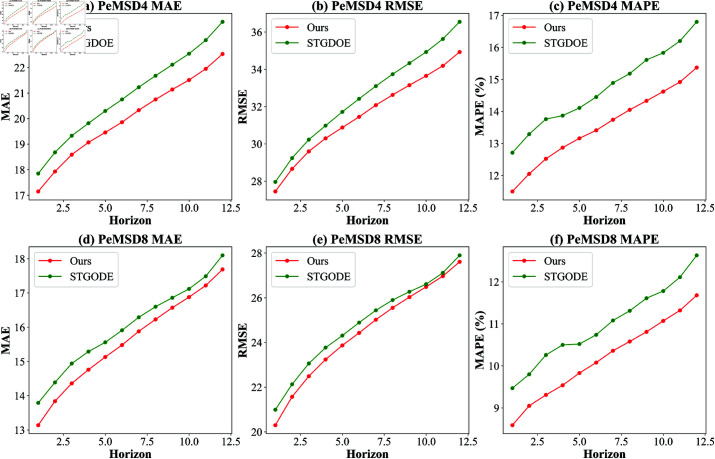
Prediction for each time slice on PeMSD4 and PeMSD8 datasets.

Table 2 and Fig 7 show the comparison results between the proposed model and 12 other prediction models on two datasets. Overall, the proposed model has the smallest prediction error on the PeMSD4 and PeMSD8 datasets.

**Table 2:** Performance comparison on PeMSD4 and PeMSD8 datasets using MAE, RMSE, and MAPE metrics.

Methods	PeMSD4			PeMSD8		
	MAE	RMSE	MAPE (%)	MAE	RMSE	MAPE (%)
HA	38.03	59.24	27.88	34.86	59.24	27.88
ARIMA	33.73	48.80	24.18	31.09	44.32	22.73
VAR	24.54	38.61	17.24	19.19	29.81	13.10
SVR	28.70	44.56	19.20	23.25	36.16	14.64
LSTM	26.77	40.65	18.23	23.09	35.17	14.99
GWN	24.89	39.66	17.29	18.28	30.05	12.15
ASTGCN(r)	22.93	35.22	16.56	18.25	28.06	11.64
STSGCN	21.19	33.65	13.90	17.13	26.80	10.96
STFGRNN	20.48	32.51	16.77	16.94	26.25	10.60
STGODE	20.93	32.57	14.73	16.03	24.87	10.99
Ours	**20.02**	**31.66**	**13.54**	**15.60**	**24.56**	**10.19**

The results show that deep learning-based methods outperform traditional statistical models (such as HA, ARIMA, and VAR), demonstrating their effectiveness in modeling highly nonlinear traffic flow. As shown in Table 2, the prediction performance of traditional methods is relatively poor. GWN models spatial dependencies in road networks using a predefined adjacency matrix, capturing spatiotemporal dependencies. Its performance surpasses that of the LSTM model, which only considers temporal dependencies, further emphasizing the importance of spatial dependencies. However, its prediction performance heavily depends on the quality of the predefined adjacency matrix.

The performance of ASTGCN and STSGCN surpasses that of earlier methods, indicating their effectiveness in capturing dynamic relationships between traffic time series. STFGNN and STGODE outperform other graph-based methods due to the introduction of temporal graphs and GODE, which expand the spatial receptive field.

As shown in Fig 7, when comparing the prediction errors of our model with the STGODE model at each time step, it can be seen that our model exhibits the smallest cumulative error. This is due to its non-end-to-end training approach: first, the multi-scale temporal attention module and multi-scale temporal convolution module are used to extract multi-scale temporal correlations, and then the graph learning module adaptively learns the spatial graph structure of the road network, leading to significant performance improvements in both short-term and long-term forecasting tasks.

### Model analysis

**Fig 8:**
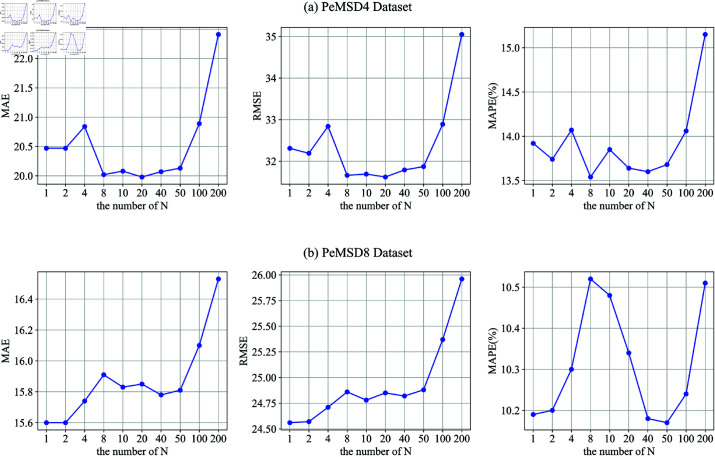
(a) Hyperparameters setting on PeMSD4. (b) Hyperparameters setting on PeMSD8.

1. Hyperparameter Settings: The total number of training epochs *M*, and the number of training epochs for the prediction module and graph learning module, *N*, have a significant impact on the results. Therefore, this section tests the effect of *M* and *N* on model performance. To ensure fairness in the experiment, the total training epochs of M*N are fixed at 200. The following figure shows the impact of different values of *N* on the results.
  From the results of the PeMSD4 dataset in Fig 8a, when the number of training epochs for the prediction module and graph learning module, *N*, is less than 50, the model performs well. However, when *N* exceeds 50, the model performance begins to decline. In our approach, the prediction module and the graph learning module adopt Alternating Optimization, a Non-End-to-End optimization strategy similar to Block Coordinate Descent (BCD). Experimental results show that when the alternating step size *N* is too large, the number of optimization steps per alternation decreases, leading to poorer convergence of the optimization process. This phenomenon does not imply that the non-end-to-end optimization method is ineffective but rather suggests that the alternating step size *N* should be moderate to ensure that each module can adequately adapt to the updates of the other module. The experimental results validate that an appropriate choice of *N* (e.g., *N* = 8 on PeMSD4) can significantly improve model performance.
  Fig 8b shows the results for the PeMSD8 dataset, with an overall trend similar to that of PeMSD4, due to the same reasons. Since the number of graph nodes in the PeMSD8 dataset is smaller, the model reaches the best training state when N=1.
2. Ablation experiments: This section aims to validate the effectiveness of different components on model performance through a series of experiments. The following variations demonstrate the performance of different module combinations:
  - w/o MTAN: In this setting, the multi-scale temporal attention module is replaced with a self-attention module to assess its impact on model performance.
  - w/o AGL: Here, the adaptive graph learning algorithm is replaced with a predefined adjacency matrix to analyze its contribution to capturing spatial relationships.
  - w/o MTCN: A single-scale temporal convolution replaces the multi-scale temporal convolution, aiding in understanding its role in temporal feature extraction.
  - w/o MTAN, AGL: This configuration uses a self-attention module and a predefined adjacency matrix in place of the multi-scale temporal attention module and adaptive graph learning, respectively, to study performance without advanced temporal attention and graph learning mechanisms.
  - w/o MTAN, MTCN: The self-attention module and single-scale temporal convolution replace the multi-scale modules to assess the overall impact of removing multi-scale capabilities.
  - w/o AGL, MTCN: A predefined adjacency matrix and a single-scale temporal convolution are used instead of adaptive graph learning and multi-scale temporal convolution, exploring performance with a fixed graph structure and single temporal scale.
  - w/o MTAN, AGL, MTCN: In this simplified model version, the multi-scale temporal attention module, adaptive graph learning algorithm, and multi-scale temporal convolution are all replaced, evaluating the comprehensive impact of minimizing complexity on model performance.

Table 3 show the ablation experiment results on the PeMSD4 and PeMSD8 datasets, respectively. From the results, it can be observed that removing a single module, particularly w/o AGL, significantly affects model performance, indicating that the predefined graph structure cannot effectively reflect the real road topology. To verify the role of multi-scale temporal information, observing the results of w/o MTAN, MTCN reveals the significant contribution of multi-scale temporal information to model performance. The overall ablation experiment results show that each module in the model improves prediction performance, proving the effectiveness and importance of the proposed modules.

**Table 3:** Ablation experiments on PeMSD4 and PeMSD8.

Selected Components			PeMSD4			PeMSD8		
MTAM	MTCN	AGL	MAE	MAPE(%)	RMSE	MAE	MAPE(%)	RMSE
			20.49	14.22	32.18	16.02	10.42	25.14
			22.53	15.58	35.25	16.87	10.85	26.47
			22.63	15.28	35.40	17.33	11.10	27.33
			22.58	15.46	35.16	17.41	11.25	27.29
			20.81	14.30	32.57	16.04	10.89	25.27
			20.51	14.05	32.16	15.81	10.29	24.79
			22.93	15.30	35.88	17.69	11.40	27.97
			**20.02**	**13.54**	**31.66**	**15.60**	**10.19**	**24.56**

### Visualization

**Fig 9:**
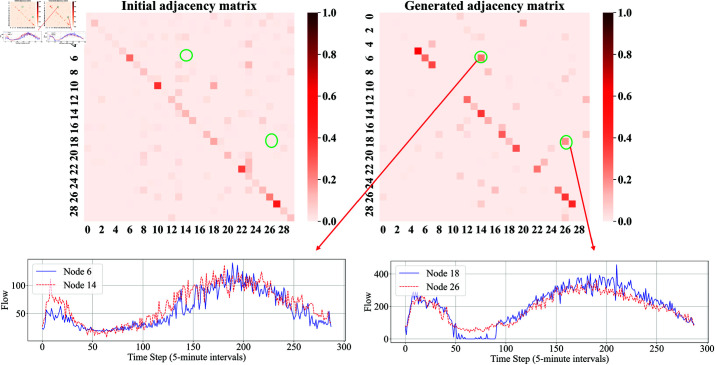
Graphical comparison of the PeMSD4 dataset. The top left shows the initial adjacency matrix, the top right displays the optimal adjacency matrix produced-by our method, and the bottom left and bottom right show the flow charts corresponding to the sensors associated with the newly generated spatial relationships.

To demonstrate the accuracy of the adjacency matrix generated by the model, the adjacency matrix diagram of 30 nodes from PeMSD4 is plotted, as shown in Fig 9. The left image is the initial adjacency matrix of the model, while the right image is the generated adjacency matrix after training.

The initial adjacency matrix in the left image shows some elements with high values, particularly concentrated along the diagonal and nearby, reflecting strong self-relations of nodes and tight connections among some local nodes. However, most other elements are close to zero, indicating relatively sparse connections between nodes.

The generated adjacency matrix in the right image shows that although the maximum value in the matrix has decreased, more elements have non-zero values, indicating that more new connections have been formed between nodes. The overall distribution of the generated adjacency matrix is more uniform, reflecting that the system learned to add new associations during training, thereby increasing the overall density of the matrix, making the relationships between nodes more complex and diverse.

To verify the rationality of the new connections in the generated adjacency matrix for the PeMSD4 dataset, a visualization analysis of two connections with larger weights in the generated matrix was conducted. The lower-left image shows the flow trends of nodes 6 and 14, and the lower-right image shows the flow trends of nodes 18 and 26. It can be seen that the flow variations between these nodes are very similar, thus the newly generated connections can be considered reasonable and effective.

**Fig 10:**
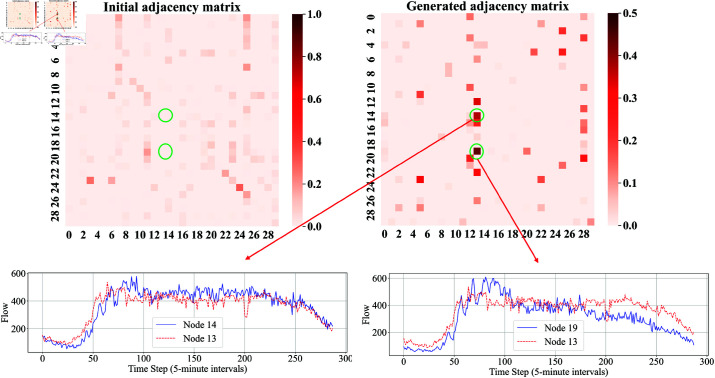
Graphical comparison of the PeMSD8 dataset. The top left shows the initial adjacency matrix, the top right displays the optimal adjacency matrix produced-by our method, and the bottom left and bottom right show the flow charts corresponding to the sensors associated with the newly generated spatial relationships.

As shown in Fig 10, the adjacency matrix diagram of 30 nodes from PeMSD8 is plotted as an example. The upper-left image is the initial adjacency matrix of the model, while the upper-right image is the generated adjacency matrix after training.

In comparison, the initial adjacency matrix shows some elements with large values (close to 1.0), indicating higher weights in certain locations, while most of the other elements have smaller values. In the generated adjacency matrix, although the maximum weight value decreased (to about 0.4), more elements have non-zero values, resulting in a more uniform distribution, indicating that more connections were introduced in the generated matrix. The initial adjacency matrix has fewer non-zero elements, reflecting strong connections between some nodes in the system, but almost no connections between most nodes. The generated adjacency matrix is denser, indicating that the system generated more weak connections, increasing the overall density of the matrix.

To verify the rationality of the new connections in the generated adjacency matrix for the PeMSD8 dataset, a visualization analysis of two connections with larger weights in the generated matrix was conducted. The lower-left image shows the flow trends of nodes 13 and 14, and the lower-right image shows the flow trends of nodes 13 and 19. It can be seen that the flow variations between these nodes are very similar, thus the newly generated connections can be considered reasonable and effective.

**Fig 11:**
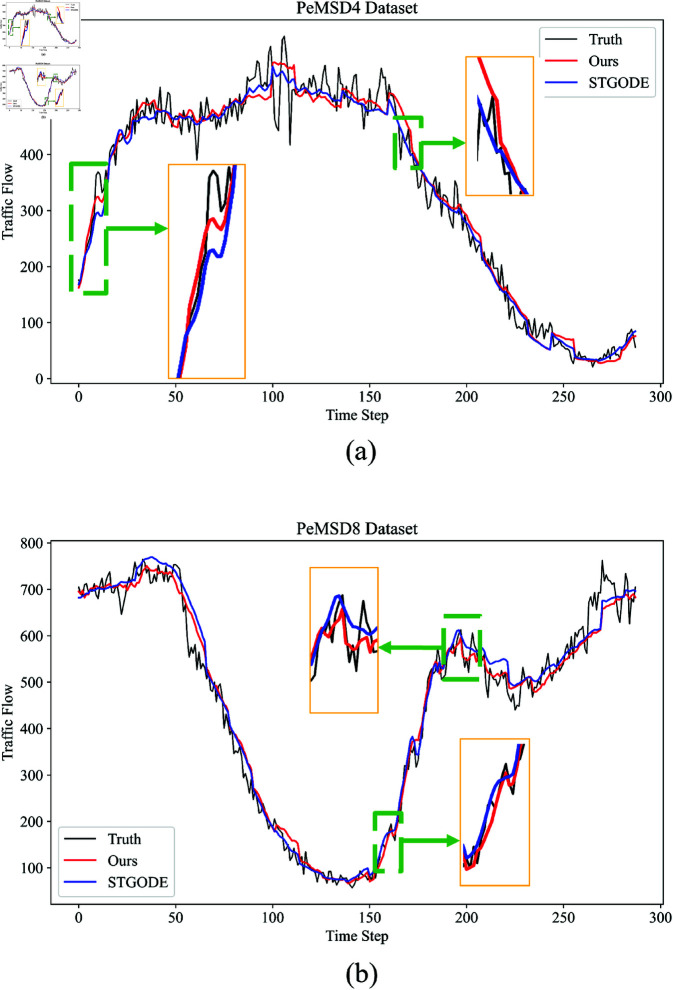
(a) The visualization of the true values and the predictions from ours model and other models on the PeMSD4. (b) The visualization of the true values and the predictions from ours model and other models on the PeMSD8.

To demonstrate the accuracy of the model’s predictions, this paper presents a case study using the PeMSD4 and PeMSD8 datasets. As shown in Fig 11, the black line represents the actual traffic flow data, the red line represents the traffic flow predicted by the proposed model, and the blue line represents the traffic flow predicted by the STGODE algorithm. From the figure, it can be seen that the trend prediction curve of the proposed model (green rectangular section) is more accurate than that of the STGODE algorithm. Therefore, the predictions of the proposed model can be considered reasonable and effective.

## Conclusion

The proposed non-end-to-end adaptive graph learning algorithm effectively overcomes the limitations of existing methods. By introducing multi-scale temporal attention and convolution modules, it successfully extracts multi-scale temporal information, enhancing the understanding of traffic states. The innovative adaptive graph learning module reveals deeper inter-node correlations during training, accurately reflecting complex road network topologies. Using non-end-to-end training with alternating optimization of prediction and graph learning module parameters, it improves each module from its local optimum, significantly enhancing predictive performance. Experimental results on the PeMSD4 and PeMSD8 datasets confirm the method’s superior performance, significantly boosting traffic flow prediction accuracy. Ablation studies further validate each module’s effectiveness. Visualizations of the dynamic graph structures generated by the graph learning module rationally reflect real traffic associations in road networks and intuitively present overall traffic flow prediction curves. These findings demonstrate the method’s significant advantage in handling complex spatiotemporal dependencies, providing crucial technical support for future intelligent transportation systems development. Although the proposed adaptive graph learning module effectively models complex road network topologies, it has not yet achieved dynamic updates of the adjacency matrix to capture instantaneous traffic variations. In addition, this study mainly focuses on short-term traffic prediction using the PeMS dataset, and the model’s generalization ability for long-term forecasting remains limited. Future work may explore the incorporation of dynamic graph modeling mechanisms and further enhance long-term prediction performance.
